# Prevalence of Iron Deficiency Anemia Indicated for Intravenous Iron Treatment in the Korean Population

**DOI:** 10.3390/nu15030614

**Published:** 2023-01-25

**Authors:** Rihwa Choi, Gayoung Chun, Mi-Jung Park, Sang Gon Lee, Eun Hee Lee

**Affiliations:** 1 Department of Laboratory Medicine, Green Cross Laboratories, Yongin 16924, Republic of Korea; 2 Department of Laboratory Medicine and Genetics, Samsung Medical Center, Sungkyunkwan University School of Medicine, Seoul 06351, Republic of Korea; 3 Infectious Disease Research Center, Green Cross Laboratories, Yongin 16924, Republic of Korea; 4 Green Cross Laboratories, Yongin 16924, Republic of Korea

**Keywords:** iron deficiency anemia, intravenous iron, chronic kidney disease, Korea

## Abstract

We aimed to investigate the number and prevalence of patients indicated for intravenous iron treatment in a large Korean population using criteria based on laboratory test results in an effort to extract indirect information on the need for intravenous iron treatment. Between 1 January 2019 and 31 December 2021, a total of 83,994 Korean patients (30,499 men and 53,495 women) with a median age of 46 years (interquartile range, 30–61) were evaluated using iron deficiency anemia–associated laboratory tests and serum creatinine tests of estimated glomerular filtration rates. The overall prevalence of anemia (Hb ≤ 11 g/dL) was 16.5%, and the proportion of patients with possible chronic kidney disease who had an estimated glomerular filtration rate < 60 mL/min/1.73 m^2^ was 11.4%. The number of patients indicated for reimbursable intravenous iron treatment was higher in women than in men, higher in older patients, and higher in 2021 than in 2019 (all *p* < 0.001). The prevalence of patients indicated for reimbursable intravenous iron treatment was up to 30.0% in those ≥ 80 years of age in 2019. The results of this study provide basic knowledge about the use of iron deficiency anemia-associated laboratory tests in planning nutritional support programs using an intravenous iron formulation in Korea.

## 1. Introduction

Iron deficiency anemia (IDA) is the predominant type of anemia, affecting more than 1.2 billion people worldwide in 2016 [1,2,3]. Because IDA can cause the progression of medical conditions, including heart failure, ischemic heart disease, and hemodynamic instability, appropriate treatment is needed to improve patient outcomes [1,3]. Treatment of IDA is performed according to etiology using oral iron, intravenous (IV) iron, and/or red blood cell transfusions (for selective patients with severe IDA who are actively bleeding or have hemodynamic symptoms, such as dyspnea, chest pain, or other relevant conditions) [1,3,4]. Oral iron supplementation is an inexpensive and effective treatment in stable patients; however, there are clinical situations for which oral iron is contraindicated, ineffective, or not tolerated, indicating the need for additional treatment options [1,5,6]. Historically, IV iron treatment was limited due to concerns about adverse effects, including severe hypersensitivity reactions, availability, accessibility, and affordability [1,3,7,8]. Improved safety and the advantage of a rapid effect with low gastrointestinal toxicity and high efficacy were notable in specific cases, such as chronic kidney disease (CKD) and inflammatory bowel disease, and in patients treated with erythropoiesis-stimulating agents, which supports the use of IV iron treatment as a personalized therapeutic approach to iron deficiency [3,5,6,9,10].

Clinical guidelines for the diagnosis and treatment of IDA recommend the use of multiple biomarkers, including hemoglobin (Hb), serum ferritin, serum iron, transferrin saturation (TSAT), serum total iron binding capacity (TIBC), and unsaturated iron binding capacity (UIBC) [1,5,6,9,11,12]. Therapeutic options other than oral supplements can be chosen according to the etiology, clinical situation, and laboratory findings of these biomarkers [1,3,7].

In Korea, IV iron formulation is reimbursed by the Health Insurance Review and Assessment Service (HIRA) only when specific conditions are met (the indication-linked reimbursement policy restricts prescriptions by medical specialists and grants reimbursement only for a subset of confirmed indications) [13]. The reimbursable conditions for use of IV iron treatment in Korea have gradually changed with regard to the criteria for Hb, serum ferritin, and TSAT. The reimbursable conditions that have been accepted since 8 May 2020, are as follows. For the general population without CKD, patients must have a recorded Hb level ≤ 10 g/dL (≤ 11 g/dL for pregnant women) and be in need of rapid iron replacement and unresponsiveness to or intolerance of oral iron replacement therapy with serum ferritin < 30 ng/mL or TSAT < 20% and occur preoperatively or immediately preceding labor. For CKD patients not receiving dialysis treatment, patients must have an Hb level ≤ 10 g/dL and serum ferritin < 100 ng/mL or TSAT < 20% to be reimbursed for IV iron formulations. Reimbursement is allowed for CKD patients on hemodialysis treatment with Hb ≤ 11 g/dL and serum ferritin < 200 ng/mL or TSAT < 20%, and for CKD patients on peritoneal dialysis with Hb ≤ 11 g/dL and serum ferritin < 100 ng/mL or TSAT < 20%. Other reimbursable cases are those receiving chemotherapy for non-myeloid malignancies; patients with Hb ≤ 10 g/dL, serum ferritin < 100 ng/mL, or TSAT < 20%; patients who are unresponsive to erythropoietin treatment for CKD or non-myeloid malignancies, with serum ferritin < 300 ng/mL or TSAT < 30% and with Hb ≤ 11 g/dL for CKD patients on dialysis and Hb ≤ 10 g/dL for patients on chemotherapy with non-myeloid malignancies. In Korea, IV iron treatment cannot be applied to patients without certain IDA-associated laboratory results due to restrictions imposed by the government payer. The number of patients who meet laboratory test results criteria can provide a broad range of information on the number and prevalence of indicated patients in Korea.

According to a factsheet published by the KoreaN Cohort Study for Outcomes in Patients With Chronic Kidney Disease (KNOW-CKD), a Korean national study of CKD patients established with support from the Korea Center for Disease Control and Prevention, IDA in CKD has increased, as evidenced by the increase in severity of CKD grade [14,15,16]. In a previous study, the proportion of iron treatment was 22.2% in CKD patients, and the rate of treatment with IV iron formulation was 7.9% among patients in the 2011–2016 KNOW-CKD cohort [14,15,16].

However, limited data are available on the prevalence of IDA in patients indicated for IV iron treatment due to changes in the reimbursement restrictions in Korea. Furthermore, recent information about the annual prevalence of IDA and patients who have possible indications for IV iron treatment is also limited. Therefore, this study investigated the prevalence of IDA and identified patients eligible for IV iron treatment by sex, age, and year. This basic knowledge of IDA will help public health agencies plan nutritional support programs using IV iron formulations in Korea.

## 2. Materials and Methods

### 2.1. Study Subjects

We retrospectively reviewed laboratory data associated with IDA and information for indicators of reimbursable IV iron treatment through the laboratory information system of Green Cross Laboratories between 1 January 2019 and 31 December 2021. Green Cross Laboratories is a referral laboratory that provides specimen analysis for IDA as requested by local clinics and hospitals in Korea. Data for Hb, serum ferritin, serum iron, UIBC, TIBC, TSAT, serum creatinine, and estimated glomerular filtration rate (eGFR) based on serum creatinine level were anonymized and analyzed. Records missing data on age or sex were excluded. 

### 2.2. Definitions

Calculations of eGFR were used to assess whether each patient had CKD. This disease was diagnosed in patients who had been tested for serum creatinine and was categorized based on eGFR < 60 mL/min/1.73 m^2^ using the Chronic Kidney Disease Epidemiology Collaboration (CKD-EPI) 2021 equation and standardized serum creatinine level, age, and sex, regardless of race [17,18].

Because the aim of this study was to provide information about the prevalence of patients possibly indicated for IV iron treatment, patients were categorized into four groups based on Hb, serum ferritin, and TSAT according to the reimbursable conditions of Korea. Group 1 comprised patients with Hb ≤ 10 g/dL and either serum ferritin < 30 ng/mL or TSAT < 20%, which could include the general population with possible IV iron treatment indication. Group 2 comprised possible CKD patients with eGFR < 60 mL/min/1.73 m^2^ and Hb ≤ 10 g/dL and either serum ferritin < 100 ng/mL or TSAT < 20%. Group 3 comprised patients with possible CKD whose eGFR was < 60 mL/min/1.73 m^2^ and with Hb ≤ 11 g/dL and either serum ferritin < 200 ng/mL or TSAT < 20%. Group 2 and group 3 may have included cases reimbursable for dialysis or non-myeloid malignancies with Hb ≤ 10 g/dL and either serum ferritin < 100 ng/mL or TSAT < 20%. The group 4 conditions were CKD with an eGFR < 60 mL/min/1.73 m^2^ and with Hb ≤ 11 g/dL and either serum ferritin < 300 ng/mL or TSAT < 30%. Such CKD patients are likely to be unresponsive to erythropoietin treatment. Group 4 could include patients on chemotherapy for non-myeloid malignancies and with Hb ≤ 10 g/dL.

The number of patients with samples tested for serum ferritin in Korea was determined based on several reimbursable test codes: D50 for management of IDA from the 10th revision, clinical modification of the International Statistical Classification of Diseases and Related Health Problems (ICD-10-CM), and D0522 for serum ferritin performed by immunoassays from the Electronic Data Interchange code by HIRA, Korea. The third reimbursable code was B03A of the Anatomical Therapeutic Chemical Classification System (ATC) code in the HIRA public database of the Healthcare Bigdata Hub. 

### 2.3. Analytical Methods

Serum ferritin was measured using Elecsys ferritin assay kits (Roche, Germany) standardized against the First International Standard (IS, 80/602) of the National Institute for Biological Standards and Control for ferritin (human liver) as measured on Cobas 8000 e801 analyzers (Roche, Germany) with traceability to more recent international standards (second IS 80/578 and third IS 94/572). Serum iron and UIBC were analyzed using iron Gen.2 assay kits (Roche) and unsaturated iron-binding capacity assay kits (Roche), respectively, on Cobas 8000 c702 analyzers. TIBC was calculated from serum iron and UIBC results using the equation TIBC = UIBC + serum iron. For eGFR calculations, serum creatinine was measured using creatinine Jaffe Gen. 2 assay kits (Roche) on Cobas 8000 c702 analyzers. Hb was measured using automated hematologic analyzers of the XN 9000 system (Sysmex, Kobe, Japan). During the study period, analytical methods were maintained without changes.

### 2.4. Statistical Analysis

Categorical variables are presented as numbers and percentages. Non-parametric methods were used when appropriate for continuous variables (age, Hb, serum ferritin, TIBC, TSAT, serum creatinine, and eGFR). We investigated the prevalence of possible CKD based on eGFR data (<60 mL/min/1.73 m^2^) measured during the study period. We investigated the prevalence of IDA in those indicated for IV iron supplementation by group based on Hb, serum ferritin, and TSAT test results and age, sex, and year. A *p*-value < 0.05 was considered statistically significant using SAS version 9.4 (SAS Institute, Inc., Cary, NC, USA).

## 3. Results

### 3.1. Baseline Characteristics of the Subjects

During the three-year study period, we obtained data from 83,994 Korean patients (30,499 men and 53,495 women) with a median age of 46 years (interquartile range, 30–61) who had been evaluated using IDA-associated laboratory tests (Hb, serum creatinine, serum ferritin, serum iron, UIBC, TIBC, and TSAT). Women were evaluated more frequently using IDA-associated tests compared with men (63.7% vs. 36.3%). The most prevalent population in this study was women aged 40 to 49 years. Baseline characteristics of study subjects and their laboratory data are summarized in Table 1. 

Among those studied, 69,178 (82.4%) were evaluated for iron status assessment only once, while the other 14,816 patients (17.6%) were tested at least twice during follow-up. The proportion of possible CKD patients with an eGFR < 60 mL/min/1.73 m^2^ was 11.4%.

All continuous baseline characteristics of age, Hb, serum ferritin, serum iron, UIBC, TIBC, TSAT, serum creatinine, and eGFR differed significantly between men and women (*p* < 0.001 based on a Wilcoxon rank sum test). Hb, serum ferritin, serum iron, UIBC, TIBC, TSAT, serum creatinine, and eGFR were significantly different between men and women in anemic patients with Hb ≤ 11 g/dL (all *p* < 0.001, Supplementary Table S1). All of these parameters except Hb were significantly different between men and women in anemic patients with Hb ≤ 10 g/dL (*p* for Hb > 0.05; < 0.001 for all others; Supplementary Table S2).

The prevalence of possible CKD with decreased eGFR < 60 mL/min/1.73 m^2^ among patients with anemia was 47.5% in patients with Hb ≤ 11 g/dL and 49.3% in patients with Hb ≤ 10 g/dL. The prevalence of anemia (Hb ≤ 11 g/dL and Hb ≤ 10 g/dL) and possible CKD with decreased eGFR < 60 mL/min/1.73 m^2^ differed significantly by sex, age, and tested year (all *p* < 0.001, from a chi-square test).

**Figure 1:**
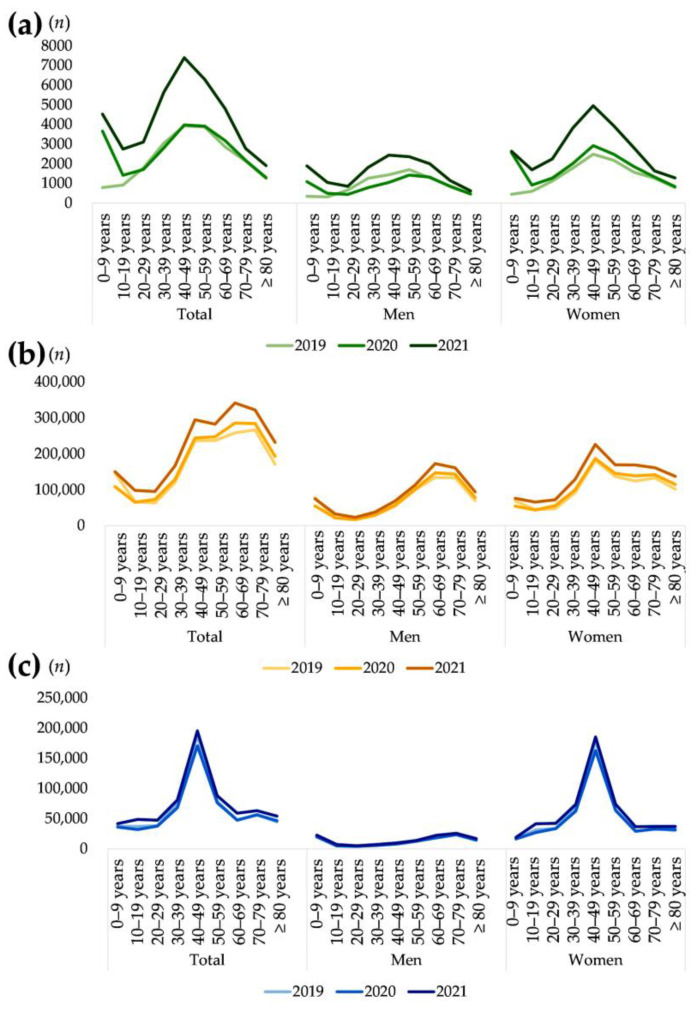
Number of patients tested for iron deficiency anemia (IDA)-associated laboratory tests by sex, age, and year. (**a**) Data of Green Cross Laboratories that performed IDA-associated laboratory tests with serum creatinine. (**b**) Data of a public database from HIRA, Korea, comprised patients who had been reimbursed for serum ferritin tests (D0522). (**c**) Data of a public database from HIRA, Korea. The *x*-axis represents age group and tested year. The *y*-axis represents the (**a**) number of patients indicated for IV iron treatment, (**b**) number of patients reimbursed for serum ferritin tests (D0522), and (**c**) number of patients who managed for IDA (D50 for ICD-10).

During the three-year study period, the number of patients evaluated using IDA-associated laboratory tests approximately doubled (from 20,620 patients in 2019 to 39,226 patients in 2021; Table 2). The number of patients who underwent IDA-associated laboratory tests was similar in 2019 and in 2020 but markedly increased in 2021 (Figure 1). Women underwent IDA-associated laboratory tests more frequently than did men during the study period, most notably in 2020. Women accounted for approximately 59.4% of cases in 2019, 67.1% in 2020, and 63.8% of total tested subjects (*p* < 0.0001). Although the number of women increased yearly, the number of men in the 20–59 year age group decreased from 2019 to 2020 and then increased by a factor of approximately two in 2021. Among men, the largest group was those aged 50–59 years in 2019 and 2020, and those aged 40–49 years in 2021.

According to monthly data from the HIRA public database, the number of patients tested for serum ferritin (D0522) gradually increased from 2019 (1,566,501 patients) to 2021 (1,984,221 patients). Women made up approximately 63.4% of all subjects in Green Cross Laboratories, a share that was comparable with data from the public database by HIRA for patients tested for serum ferritin (D0522, 60.1%). Among men, the largest group of patients was aged 60–69 years. The number of patients managed for IDA (D50) was stable from 2019 to 2021, with the largest number of patients aged 40 to 49 years (Figure 1).

### 3.2. Prevalence of Patients Indicated for IV Iron Treatment

**Figure 2:**
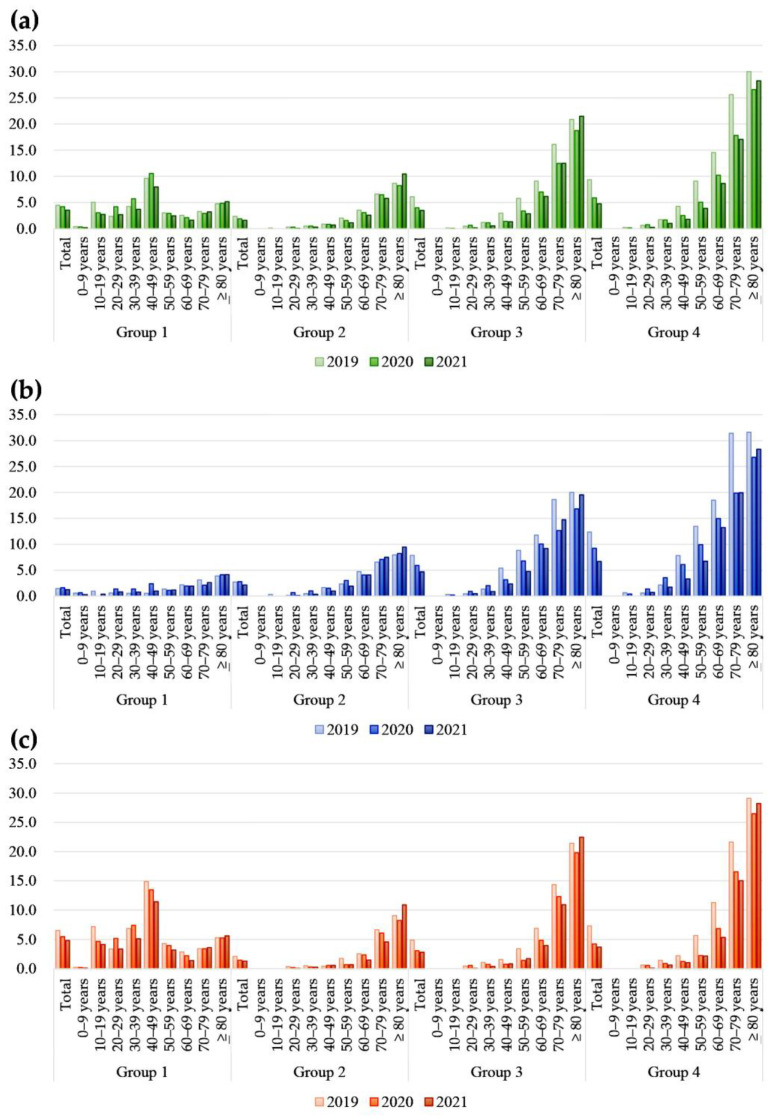
Prevalence of patients indicated for IV iron treatment by sex, age, and year (%) for (**a**) total subjects, (**b**) men, and (**c**) women. The *x*-axis represents age and tested year. The *y*-axis represents the percentage of patients indicated for reimbursable IV iron treatment. Group 1 was patients with eGFR ≥ 60 mL/min/1.73 m^2^, Hb ≤ 10 g/dL, and either serum ferritin < 30 ng/mL or TSAT < 20%. Group 2 was patients with eGFR < 60 mL/min/1.73 m^2^, Hb ≤ 10 g/dL, and either serum ferritin < 100 ng/mL or TSAT < 20%. Group 3 was those with eGFR < 60 mL/min/1.73 m^2^, Hb ≤ 11 g/dL, and either serum ferritin < 200 ng/mL or TSAT < 20%. Group 4 comprised those with eGFR < 60 mL/min/1.73 m^2^, Hb ≤ 11 g/dL, and either serum ferritin < 300 ng/mL or TSAT < 30%.

**Figure 3:**
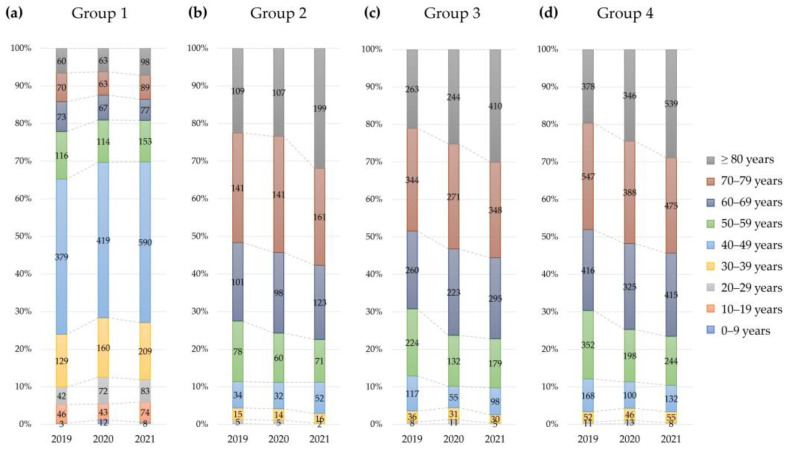
Proportion and number of patients indicated for IV iron treatment by age and tested year (**a**) Group 1 patients with eGFR ≥ 60 mL/min/1.73 m^2^, Hb ≤ 10 g/dL, and either serum ferritin < 30 ng/mL or TSAT < 20%. (**b**) Group 2 patients with eGFR < 60 mL/min/1.73 m^2^, Hb ≤ 10 g/dL, and either serum ferritin < 100 ng/mL or TSAT < 20%. (**c**) Group 3 patients with eGFR < 60 mL/min/1.73 m^2^, Hb ≤ 11 g/dL, and either serum ferritin < 200 ng/mL or TSAT < 20%. (**d**) Group 4 patients with eGFR < 60 mL/min/1.73 m^2^, Hb ≤ 11 g/dL, and either serum ferritin < 300 ng/mL or TSAT < 30%. The *x*-axis represents tested year, and the *y*-axis represents the proportion of patients by age group. Numbers in the bar graph represent the numbers of patients indicated for IV iron treatment.

The number and prevalence of patients in the four groups of reimbursable IV iron treatment conditions based on Hb and either serum ferritin or TSAT by sex, age, and year are summarized in Table 3 and Figure 2 and Figure 3. The prevalence of IDA with reimbursable IV iron treatment differed significantly by sex, age, and tested year (*p* < 0.0001).

The prevalence of IDA patients indicated for IV iron treatment was significantly different according to the group of reimbursable conditions by sex (*p* < 0.0001). For group 1, the overall prevalence of women was approximately three times higher than that of men (5.4% in women and 1.4% in men, *p* < 0.0001). The difference in prevalence for group 1 was largest in patients aged 40 to 49 years (1.2% in men and 12.8% in women). For groups 2, 3, and 4, the overall prevalence was higher in men than in women. For group 4, the prevalence of men was approximately three times higher than that in women aged 40 to 59 years, opposite the prevalence in group 1.

The prevalence of IDA patients indicated for IV iron treatment was significantly different according to group of reimbursable conditions by age (*p* < 0.0001). The overall prevalence of IDA was higher in older than younger patients. However, for the group 1 population (patients without possible CKD), the prevalence of reimbursable IV iron treatment conditions was highest in patients younger than 50 years. Approximately one-third of total patients older than 80 years met the group 4 condition. 

The prevalence of IDA indicated for reimbursable IV iron treatment differed significantly according to group of reimbursable conditions by tested year (*p* < 0.0001). For group 1 condition, the overall prevalence in patients in their teens and 60s decreased from 2019 to 2021. The prevalence in patients in the other ages in group 1 were stable, slightly increased, or fluctuated. For groups 3 and 4, the prevalence gradually decreased in patients aged 30 to 79 years. Among reimbursable conditions, patients indicated for reimbursable IV iron treatment represented up to 30.0% of those 80 years of age or older in 2019.

## 4. Discussion

We investigated the prevalence and number of IDA patients indicated for IV iron treatment in a large Korean population visiting local clinics and hospitals based on the reimbursable conditions as measured on laboratory tests for IDA.

The prevalence of IDA in this study was comparable to the global prevalence [3,19,20]. Among study subjects, most received all IDA-associated laboratory tests only once. The proportion of patients who had follow-up IDA-associated laboratory tests was similar to the proportion of anemic patients. This might suggest that physicians in Korea tend to utilize all IDA-associated laboratory tests and that they use these tests to follow selected patients [1,3]. Because certain laboratory test results are needed for IV iron treatment, patients with IDA may be monitored with those tests. 

The proportion of patients with CKD was lower than that of anemic patients overall. This suggests that the patient groups identified by laboratory tests in this study included patients with various diseases associated with IDA described in the reimbursable clinical conditions.

The prevalence of anemia with Hb ≤ 10 g/dL or Hb ≤ 11 g/dL and possible CKD with decreased eGFR < 60 mL/min/1.73 m^2^ differed significantly by sex, age, and test year. This finding was comparable to those of previous studies of IDA [3,14,16]. A higher prevalence of anemia and CKD was observed in women and elderly patients, a finding comparable to that of previous research in Korean and other populations [3,14,16].

The population included in this study was younger than that from the HIRA database. However, the largest group of patients managed for IDA using ICD-10 D50 was comparable to the number of subjects in this study at Green Cross Laboratories. Considering that Green Cross Laboratories is a referral laboratory for local clinics and hospitals without their own clinical laboratories, and that older patients and those with comorbidities visit university hospitals and medical centers more frequently than others, the differences in patient population characteristics observed in this study may be due to the healthcare-related behaviors of patients [21]. In addition, the number of patients from the HIRA database includes repeated data from some subjects, which may lead to overestimates of utilization in specific age groups. This effect may be alleviated by the variety of population characteristics within the cohort. Data for subjects in this study from Green Cross Laboratories did not include repeated data from the same subjects. 

In this study, the prevalence of CKD was assessed based on eGFR, which was estimated using the CKD-EPI 2021 calculation [17,18]. Because use of the equation has only recently increased, limited data are available for a large number of Korean patients [18,22]. In Korea, the Korea National Health and Nutrition Examination Survey (KNHANES) data, which are publicly available (https://knhanes.kdca.go.kr/knhanes/sub03/sub03_02_05.do, accessed on 16 November 2022), include only laboratory data measured up to 2020, and the prevalence of CKD using eGFR is based on the CKD-EPI 2012 equation. Previous studies performed in Korean patients who had health checkups indicate that the prevalence of CKD could vary by the equation used to assess eGFR, which requires further studies to validate the clinical implications of the new CKD-EPI 2021 calculation [18]. The overall prevalence of possible CKD with decreased eGFR < 60 mL/min/1.73 m^2^ was 11.4%, which was comparable with the KNHANES data, in which the overall prevalence of CKD in 2019 was 9.3%. 

The stable or decreasing annual percentage of patients with possible indications for IV iron treatment during the study period may be due to the large number of tested patients screened. The annual number of patients evaluated using IDA-associated laboratory tests with serum creatinine and identified as having indications for IV iron treatment increased during the study period. These findings suggest that more widespread screening using blood tests could identify more patients indicated for IV iron treatment. As information on the number of patients indicated for IV iron treatment is important for a national preparedness plan, disease prevalence and number of patients should be monitored continuously. 

According to the HIRA public database of Healthcare Bigdata Hub, the number of reimbursed iron preparations (ATC code: B03A) increased from 2019 to 2021. The most common disease treated with iron preparation (ATC code B03A) was CKD, followed by IDA. However, the number of parenteral iron supplementation cases (ATC code B03AC) was not in the database. According to a public database maintained by the Ministry of Food and Drug Safety, Korea (https://nedrug.mfds.go.kr/index, accessed on 30 November 2022), the cost of manufactured parenteral preparations of iron in Korea was approximately USD 2.4 million in 2019, USD 1.8 million in 2020, and USD 2.1 million in 2021. According to the database, imports of parenteral preparations of iron from European countries cost USD 6.1 million in 2019 and USD 9.9 million in 2020 (import cost data for 2021 were not available). Although the exact number of uses of IV iron treatment in Korea was not available by year, the increase in overall manufactured and imported parenteral iron preparations was comparable to the increase in the number of patients indicated for IV iron treatment in Korea observed in this study population. According to the Healthcare Bigdata Hub, the number of patients managed for CKD, IDA, IBD, or non-myeloid malignancies also increased from 2019 to 2021. This suggests that laboratory test results-based estimates of the number and prevalence of patients with possible IV iron treatment can produce useful estimates of the financial burden of IV iron treatment. The number of patients with these diseases and indications for IV iron treatment should be monitored to ensure sufficient stocks of IV iron formulations. A recent study in Japan reported that the amount of infused IV iron available may not be sufficient for IDA patients [19]. Future studies with comprehensive information on clinical information, the actual amount of IV iron supplements, and the laboratory test results could identify unmet needs for indicated patients.

One limitation of the present study was the lack of clinical information associated with IDA, such as comorbidities, medications, and supplement use. Iron status using a blood test can be affected by various factors. For example, serum ferritin is an acute-phase reactant that fluctuates with the clinical situation of patients with inflammatory processes and other factors [12]. CKD is a broad spectrum of disease states, and the reimbursement criteria differ based on renal replacement therapy. Information about erythropoietin treatment or malignancies was unavailable. The patient grouping was based on IDA-associated laboratory test results instead of clinical grouping, which provides only general information about overall needs without definitely matching specific groups of patients by disease and treatment type. The study period includes the coronavirus disease 2019 (COVID-19) pandemic, the effects of which on the healthcare characteristics of the study population may differ by year [23]. The results may not be generalizable to a non-pandemic situation [23]. However, IDA can be asymptomatic, and Hb, serum ferritin, and TSAT are used to categorize iron status for reimbursable IV iron treatment in Korea. The findings of this study may be generalizable to populations in regions with payer restrictions for use of IV iron treatment based on IDA-associated laboratory test results, and populations consisted of patients visiting local clinics and hospitals. The scope of this study does not include the safety of IV iron treatment, which is an important concern [3,19,24]. Future studies with comprehensive clinical information, including broad aspects of IV iron treatment such as safety, are needed to clarify the utilization of IDA-associated laboratory tests and the number and prevalence of patients with possible indications for IV iron treatment. 

The strength of the present study includes the large number of subjects studied over a long period for the analysis of the prevalence of Korean patients indicated for IV iron treatment [1,6,11]. In addition, recent data for many Koreans provided additional information about the prevalence of anemia and CKD. The results of this study could help set public health guidelines for managing IDA patients in the Korean population. Our findings suggest that laboratory data analysis using reimbursable criteria can be used to assess the possible numbers and prevalence of patients indicated for IV iron treatment. Furthermore, the stable proportion of patients indicated for IV iron treatment may help identify populations at greater risk who require IV iron treatment. The results of this study could also help strengthen understanding of IDA patients in the Korean population.

## 5. Conclusions

This study investigated the prevalence of IDA patients indicated for IV iron treatment in a large Korean population of patients visiting local clinics and hospitals. Using reimbursable conditions based on laboratory tests for IDA, different prevalence rates of patients with IDA and patients possibly indicated for IV iron treatment were observed by sex, age, and year of testing. An increase in the annual number of patients suggests that additional screening using blood tests could identify more patients indicated for IV iron treatment. The results of this study could help strengthen the understanding of IDA in the Korean population.

## Figures and Tables

**Table 1:** Baseline characteristics of 83,994 study subjects.

Characteristics	Total Subjects (*n* = 83,994)	Men (*n* = 30,499)	Women (*n* = 53,495)
Age, years (median, IQR)	46 (30–61)	48 (31–62)	45 (29–60)
Age group (*n*, %)			
0–9 years	8986 (10.7)	3329 (10.9)	5657 (10.6)
10–19 years	5085 (6.1)	1874 (6.1)	3211 (6.0)
20–29 years	6614 (7.9)	1962 (6.4)	4652 (8.7)
30–39 years	11,493 (13.7)	3872 (12.7)	7621 (14.2)
40–49 years	15,313 (18.2)	4940 (16.2)	10,373 (19.4)
50–59 years	14,089 (16.8)	5491(18.0)	8598 (16.1)
60–69 years	10,850 (12.9)	4617 (15.1)	6233 (11.7)
70–79 years	7096 (8.4)	2862 (9.4)	4234 (7.9)
≥80 years	4468 (5.3)	1552 (5.1)	2916 (5.5)
Follow-up, number (median, IQR, min–max)	1 (1–1, 1–40)	1 (1–1, 1–40)	1 (1–1, 1–36)
Baseline laboratory test result			
Hb, g/dL (median, IQR)	13.1 (11.9–14.3)	14.4 (12.4–15.6)	12.8 (11.7–13.6)
Serum ferritin, ng/mL (median, IQR)	89 (43–192)	184 (86–319)	65 (32–119)
Serum iron, ug/dL (median, IQR)	93 (64–123)	100 (71–132)	89 (61–117)
UIBC, ug/dL (median, IQR)	215 (171–264)	197 (155–242)	224 (181–278)
TIBC, ug/dL (median, IQR)	316 (280–356)	307 (270–343)	322 (286–363)
TSAT, % (median, IQR)	29.8 (21.1–39.6)	32.8 (24.1–43.3)	28.21 (19.3–37.4)
Serum creatinine, mg/dL (median, IQR)	0.7 (0.6–0.9)	0.9 (0.8–1.1)	0.7 (0.6–0.8)
eGFR, mL/min/1.73 m^2^, (median, IQR)	104.8 (88.5–119.5)	99.44 (79.4–115.5)	107.74 (92.5–121.1)
Patients with anemia with Hb ≤ 11 g/dL (*n*, %)	13,827 (16.5)	4594 (15.1)	9233 (17.3)
Patients with anemia with Hb ≤ 10 g/dL (*n*, %)	7845 (9.3)	2700 (8.9)	5145 (9.6)
Possible CKD with eGFR < 60 mL/min/1.73 m^2^ (*n*, %)	9587 (11.4)	5165 (16.9)	4422 (8.3)

Abbreviations: CKD, chronic kidney disease; eGFR, estimated glomerular filtration rate; IQR, interquartile range; TIBC, total iron-binding capacity; TSAT, transferrin saturation; UIBC, unsaturated iron-binding capacity. Possible CKD patients were defined as eGFR < 60 mL/min/1.73 m^2^ (*n*, %).

**Table 2:** Number of patients tested for iron deficiency anemia-associated laboratory tests and serum creatinine by sex, age, and year.

Age	2019			2020			2021		
	Total (*n* = 20,620)	Men (*n* = 8364)	Women (*n* = 12,256)	Total (*n* = 24,148)	Men (*n* = 7936)	Women (*n* = 16,212)	Total (*n* = 39,226)	Men (*n* = 14,199)	Women (*n* = 25,027)
0–9 years	794	343	451	3662	1096	2566	4530	1890	2640
10–19 years	916	317	599	1421	501	920	2748	1056	1692
20–29 years	1793	667	1126	1713	438	1275	3108	857	2251
30–39 years	3048	1265	1783	2802	788	2014	5643	1819	3824
40–49 years	3935	1445	2490	3977	1052	2925	7401	2443	4958
50–59 years	3877	1702	2175	3914	1429	2485	6298	2360	3938
60–69 years	2862	1291	1571	3181	1324	1857	4807	2002	2805
70–79 years	2136	869	1267	2176	845	1331	2784	1148	1636
≥80 years	1259	465	794	1302	463	839	1907	624	1283

**Table 3:** Prevalence of patients indicated for IV iron treatment by sex, age, and year.

Group	Age	2019						2020						2021					
		Total (*n* = 20,620)		Men (*n* = 8364)		Women (*n* = 12,256)		Total (*n* = 24,148)		Men (*n* = 7936)		Women (*n* = 16,212)		Total (*n* = 39,226)		Men (*n* = 14,199)		Women (*n* = 25,027)	
		*n*	%	*n*	%	*n*	%	*n*	%	*n*	%	*n*	%	*n*	%	*n*	%	*n*	%
**Group 1** eGFR ≥ 60 mL/min/1.73 m^2^ and Hb ≤ 10 g/dL and Serum ferritin <30 ng/mL or TSAT < 20%	Total	918	4.5	120	1.4	798	6.5	1013	4.2	128	1.6	885	5.5	1381	3.5	176	1.2	1205	4.8
	0–9 years	3	0.4	2	0.6	1	0.2	12	0.3	7	0.6	5	0.2	8	0.2	5	0.3	3	0.1
	10–19 years	46	5.0	3	0.9	43	7.2	43	3.0	0	0.0	43	4.7	74	2.7	4	0.4	70	4.1
	20–29 years	42	2.3	4	0.6	38	3.4	72	4.2	6	1.4	66	5.2	83	2.7	7	0.8	76	3.4
	30–39 years	129	4.2	7	0.6	122	6.8	160	5.7	11	1.4	149	7.4	209	3.7	14	0.8	195	5.1
	40–49 years	379	9.6	8	0.6	371	14.9	419	10.5	25	2.4	394	13.5	590	8.0	24	1.0	566	11.4
	50–59 years	116	3.0	23	1.4	93	4.3	114	2.9	16	1.1	98	3.9	153	2.4	28	1.2	125	3.2
	60–69 years	73	2.6	28	2.2	45	2.9	67	2.1	26	2.0	41	2.2	77	1.6	38	1.9	39	1.4
	70–79 years	70	3.3	27	3.1	43	3.4	63	2.9	18	2.1	45	3.4	89	3.2	30	2.6	59	3.6
	≥80 years	60	4.8	18	3.9	42	5.3	63	4.8	19	4.1	44	5.2	98	5.1	26	4.2	72	5.6
**Group 2** eGFR < 60 mL/min/1.73 m^2^ and Hb ≤ 10 g/dL and Serum ferritin < 100 ng/mL or < TSAT 20%	Total	484	2.3	227	2.7	257	2.1	457	1.9	222	2.8	235	1.4	624	1.6	303	2.1	321	1.3
	0–9 years	0	0.0	0	0.0	0	0.0	0	0.0	0	0.0	0	0.0	0	0.0	0	0.0	0	0.0
	10–19 years	1	0.1	1	0.3	0	0.0	0	0.0	0	0.0	0	0.0	0	0.0	0	0.0	0	0.0
	20–29 years	5	0.3	1	0.1	4	0.4	5	0.3	3	0.7	2	0.2	2	0.1	1	0.1	1	0.0
	30–39 years	15	0.5	6	0.5	9	0.5	14	0.5	8	1.0	6	0.3	16	0.3	6	0.3	10	0.3
	40–49 years	34	0.9	24	1.7	10	0.4	32	0.8	16	1.5	16	0.5	52	0.7	24	1.0	28	0.6
	50–59 years	78	2.0	40	2.4	38	1.7	60	1.5	43	3.0	17	0.7	71	1.1	45	1.9	26	0.7
	60–69 years	101	3.5	61	4.7	40	2.5	98	3.1	54	4.1	44	2.4	123	2.6	82	4.1	41	1.5
	70–79 years	141	6.6	57	6.6	84	6.6	141	6.5	60	7.1	81	6.1	161	5.8	86	7.5	75	4.6
	≥80 years	109	8.7	37	8.0	72	9.1	107	8.2	38	8.2	69	8.2	199	10.4	59	9.5	140	10.9
**Group 3** eGFR < 60 mL/min/1.73 m^2^ and Hb ≤ 11 g/dL and Serum ferritin <200 ng/mL or < TSAT 20%	Total	1253	6.1	656	7.8	597	4.9	968	4.0	469	5.9	499	3.1	1365	3.5	664	4.7	701	2.8
	0–9 years	0	0.0	0	0.0	0	0.0	0	0.0	0	0.0	0	0.0	0	0.0	0	0.0	0	0.0
	10–19 years	1	0.1	1	0.3	0	0.0	1	0.1	1	0.2	0	0.0	0	0.0	0	0.0	0	0.0
	20–29 years	8	0.4	3	0.4	5	0.4	11	0.6	4	0.9	7	0.5	5	0.2	4	0.5	1	0.0
	30–39 years	36	1.2	17	1.3	19	1.1	31	1.1	16	2.0	15	0.7	30	0.5	16	0.9	14	0.4
	40–49 years	117	3.0	78	5.4	39	1.6	55	1.4	33	3.1	22	0.8	98	1.3	57	2.3	41	0.8
	50–59 years	224	5.8	150	8.8	74	3.4	132	3.4	97	6.8	35	1.4	179	2.8	112	4.7	67	1.7
	60–69 years	260	9.1	152	11.8	108	6.9	223	7.0	133	10.0	90	4.8	295	6.1	184	9.2	111	4.0
	70–79 years	344	16.1	162	18.6	182	14.4	271	12.5	107	12.7	164	12.3	348	12.5	169	14.7	179	10.9
	≥80 years	263	20.9	93	20.0	170	21.4	244	18.7	78	16.8	166	19.8	410	21.5	122	19.6	288	22.4
**Group 4** eGFR < 60 mL/min/1.73 m^2^ and Hb ≤ 11 g/dL and Serum ferritin <300 ng/mL or < TSAT 30%	Total	1926	9.3	1034	12.4	892	7.3	1418	5.9	732	9.2	686	4.2	1869	4.8	949	6.7	920	3.7
	0–9 years	0	0.0	0	0.0	0	0.0	0	0.0	0	0.0	0	0.0	0	0.0	0	0.0	0	0.0
	10–19 years	2	0.2	2	0.6	0	0.0	2	0.1	2	0.4	0	0.0	0	0.0	0	0.0	0	0.0
	20–29 years	11	0.6	4	0.6	7	0.6	13	0.8	6	1.4	7	0.5	8	0.3	6	0.7	2	0.1
	30–39 years	52	1.7	27	2.1	25	1.4	46	1.6	28	3.6	18	0.9	55	1.0	31	1.7	24	0.6
	40–49 years	168	4.3	113	7.8	55	2.2	100	2.5	64	6.1	36	1.2	132	1.8	81	3.3	51	1.0
	50–59 years	352	9.1	229	13.5	123	5.7	198	5.1	142	9.9	56	2.3	244	3.9	159	6.7	85	2.2
	60–69 years	416	14.5	239	18.5	177	11.3	325	10.2	198	15.0	127	6.8	415	8.6	265	13.2	150	5.3
	70–79 years	547	25.6	273	31.4	274	21.6	388	17.8	168	19.9	220	16.5	475	17.1	229	19.9	246	15.0
	≥80 years	378	30.0	147	31.6	231	29.1	346	26.6	124	26.8	222	26.5	539	28.3	177	28.4	362	28.2

Abbreviations: eGFR, estimated glomerular filtration rate; TSAT, transferrin saturation. Possible CKD patients were defined when eGFR < 60 mL/min/1.73 m^2^. Group 1 patients with eGFR ≥ 60 mL/min/1.73 m^2^, Hb ≤ 10 g/dL, and either serum ferritin < 30 ng/mL or TSAT < 20%. Group 2 patients with eGFR < 60 mL/min/1.73 m^2^, Hb ≤ 10 g/dL, and either serum ferritin < 100 ng/mL or TSAT < 20%. Group 3 patients with eGFR < 60 mL/min/1.73 m^2^, Hb ≤ 11 g/dL, and either serum ferritin < 200 ng/mL or TSAT < 20%. Group 4 patients with eGFR < 60 mL/min/1.73 m^2^, Hb ≤ 11 g/dL, and either serum ferritin < 300 ng/mL or TSAT < 30%.

## Data Availability

The datasets generated and analyzed during the current study are available from the corresponding authors on reasonable request.

## Supplementary Materials

The following supporting information can be downloaded at: https://www.mdpi.com/article/10.3390/nu15030614/s1, Table S1: Laboratory test results in anemic patients with Hb ≤ 11 g/dL, Table S2: Laboratory test results in anemic patients with Hb ≤ 10 g/dL.
