# Supplementary material for: Prevalence of Iron Deficiency Anemia Indicated for Intravenous Iron Treatment in the Korean Population
Source: Nutrients. 2023 Jan 25;15(3):614. doi: 10.3390/nu15030614 (PMC9919267; doi:10.3390/nu15030614)
Supplement: Supplementary file 1 [file nutrients-15-00614-s001.zip › nutrients-2140845-supplementary.pdf]

Prevalence of iron deficiency anemia indicated for intravenous iron treatment in the Korean population

Supplementary Table S1. Laboratory test results in anemic patients with Hb ≤ 11 g/dL.

Characteristics	Total subjects (n = 13,827)	Men (n = 4,594)	Women (n = 9,233)	p-value of Wilcoxon rank-sum test
Hb, g/dL	9.8 (8.9 to 10.5)	9.8 (8.8 to 10.5)	9.9 (8.9 to 10.5)	0.0003
Serum ferritin, ng/mL	85.0 (10.0 to 283.0)	212.0 (82.0 to 435.0)	33.0 (7.0 to 185.0)	< 0.0001
Serum iron, ug/dL	46.0 (27.0 to 72.0)	54.0 (36.0 to 77.0)	41.0 (24.0 to 69.0)	< 0.0001
UIBC, ug/dL	213.0 (148.0 to 350.0)	163.0 (125.0 to 215.0)	276.0 (170.0 to 375.0)	< 0.0001
TIBC, ug/dL	281.0 (214.0 to 386.0)	228.0 (191.0 to 276.0)	335.0 (239.0 to 406.0)	< 0.0001
TSAT, %	19.1 (7.9 to 30.2)	24.9 (16.6 to 35.1)	14.8 (6.3 to 27.1)	< 0.0001
Serum creatinine, mg/dL	1.00 (0.68 to 6.10)	6.00 (1.30 to 9.50)	0.73 (0.60 to 1.50)	< 0.0001
eGFR, mL/min/1.73m^2	66.7 (8.3 to 108.4)	9.5 (5.7 to 57.0)	92.5 (35.2 to 112.1)	< 0.0001

Abbreviations: eGFR, estimated glomerular filtration rate; TIBC, total iron-binding capacity; TSAT, Transferrin saturation; UIBC, unsaturated iron-binding capacity. Data are presented as median and interquartile range.

Supplementary Table S2. Laboratory test results in anemic patients with Hb ≤ 10 g/dL.

Characteristics	Total subjects (n = 7,845)	Men (n = 2,700)	Women (n = 5,145)	p-value of Wilcoxon rank-sum test
Hb, g/dL	9.0 (8.2 to 9.6)	9.0 (8.2 to 9.6)	9.0 (8.2 to 9.6)	0.9707
Serum ferritin, ng/mL	83.0 (7.0 to 326.0)	237.5 (85.0 to 494.0)	19.0 (5.0 to 201.0)	< 0.0001
Serum iron, ug/dL	37.0 (21.0 to 62.0)	48.0 (31.0 to 71.0)	41.0 (20.0 to 56.0)	< 0.0001
UIBC, ug/dL	215.0 (141.0 to 370.0)	156.0 (117.0 to 215.0)	312.0 (166.0 to 393.0)	< 0.0001
TIBC, ug/dL	277.0 (202.0 to 396.0)	219.0 (179.5 to 272.5)	353.0 (231.0 to 418.0)	< 0.0001
TSAT, %	16.7 (5.9 to 28.7)	23.7 (15.0 to 34.3)	9.7 (4.9 to 24.8)	< 0.0001
Serum creatinine, mg/dL	1.02 (0.68 to 5.80)	5.54 (1.30 to 8.62)	0.71 (0.60 to 1.90)	< 0.0001
eGFR, mL/min/1.73m^2	62.3 (8.8 to 109.1)	10.5 (6.4 to 57.0)	93.6 (26.9 to 112.7)	< 0.0001

Abbreviations: eGFR, estimated glomerular filtration rate; TIBC, total iron-binding capacity; TSAT, Transferrin saturation; UIBC, unsaturated iron-binding capacity. Data are presented as median and interquartile range.
